# Supplementary figures and images for: A novel prevascularized tissue-engineered chamber as a site for allogeneic and xenogeneic islet transplantation to establish a bioartificial pancreas
Source: PLoS One. 2020 Dec 3;15(12):e0234670. doi: 10.1371/journal.pone.0234670 (PMC7714105; doi:10.1371/journal.pone.0234670)

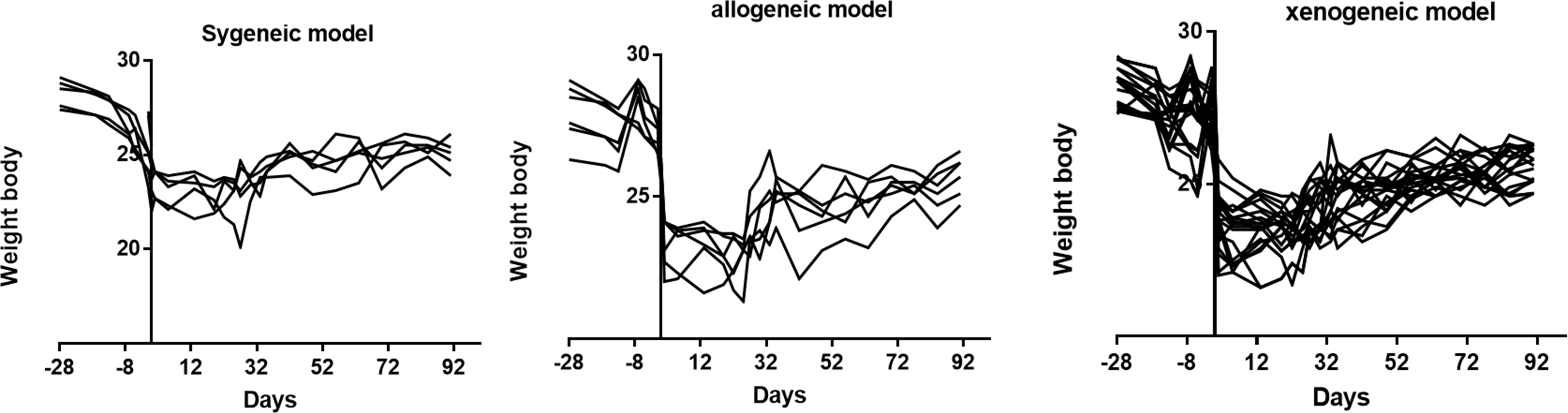

Supplement: S1 Fig — Continued weight stability indicated the overall safety of the TEC 28 d post-implantation. Weight increased after transplantation, closely correlating with treatment regimens in the TEC. (TIF) [file pone.0234670.s001.tif]

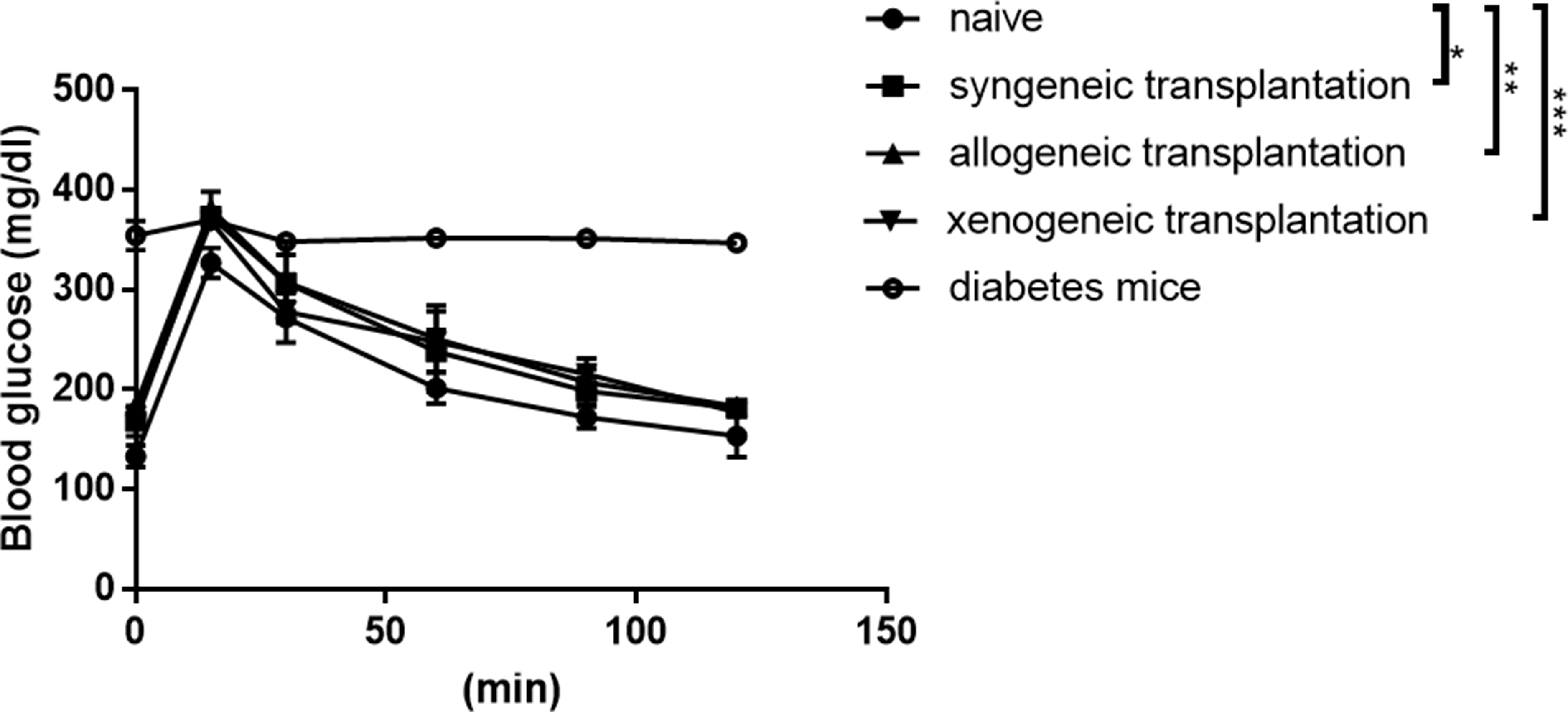

Supplement: S2 Fig — The naive mice were nondiabetic, nontransplanted C57 mice (black, n = 3), which are more tolerant of metabolic tests than transplant recipients. Blood glucose measurements were monitored at t = 0, 15, 30, 60, 90, and 120 minutes. Data points represent the mean ± S.E.M. of blood glucose values. No difference in the tolerance of mice to glucose challenge was observed in mice that received syngeneic, allogeneic, or xenogeneic islets in a TEC (n = 3) compared to naive animals (p*>0.01 vs. syngeneic group, p**>0.01 vs. allogeneic group, p***>0.01 vs. xenogeneic group). (TIF) [file pone.0234670.s002.tif]
