# Supplementary material for: A novel prevascularized tissue-engineered chamber as a site for allogeneic and xenogeneic islet transplantation to establish a bioartificial pancreas
Source: PLoS One. 2020 Dec 3;15(12):e0234670. doi: 10.1371/journal.pone.0234670 (PMC7714105; doi:10.1371/journal.pone.0234670)

Raw data———Blot

Anti-VEGF

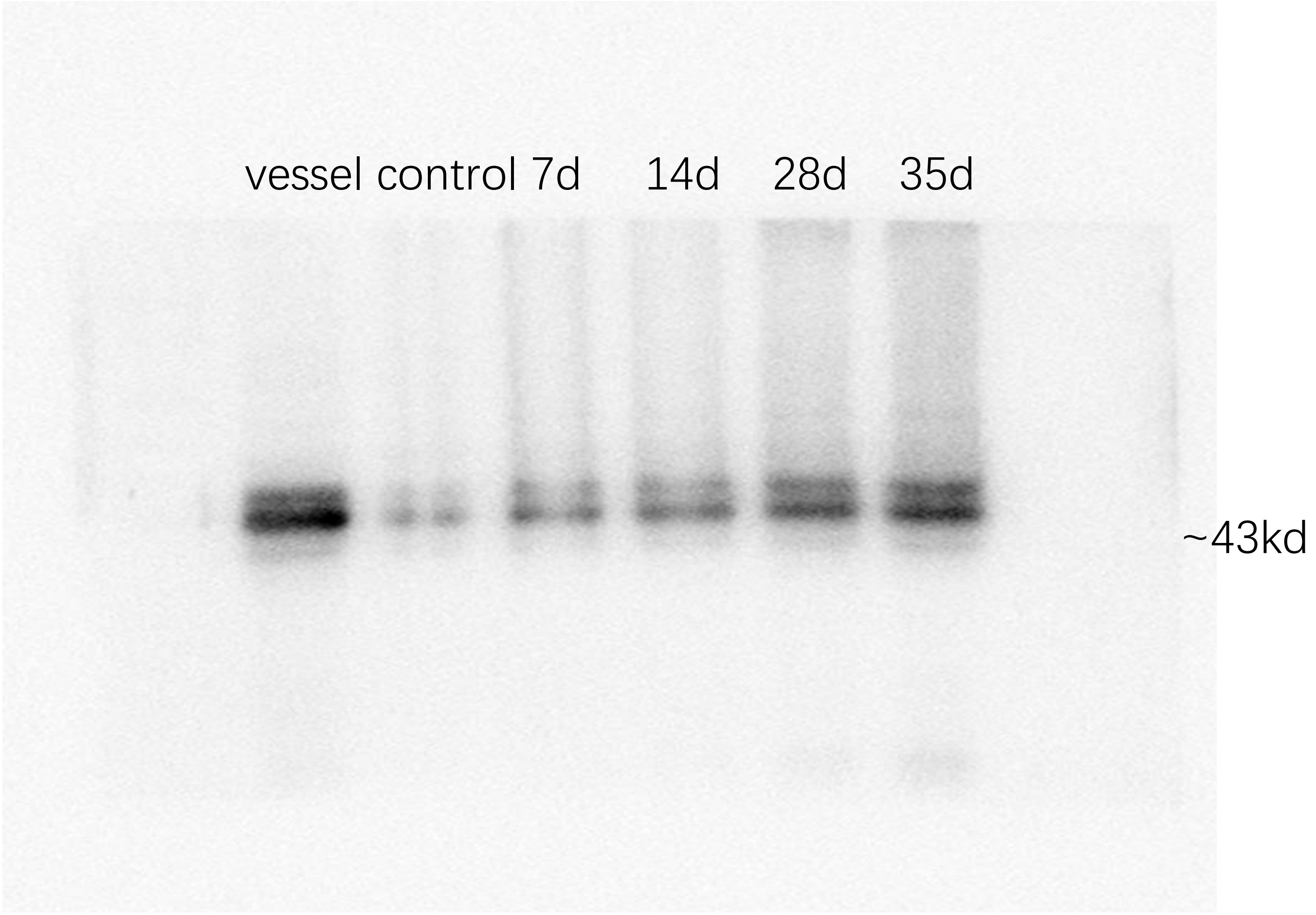

Fig2 C. Relevant data underlying the finding sdescribed in manuscript.

Sample

Vessel: abdominal vessel

Control: Fat pad

7d, 14d, 28d, 35d:sample from pre-vascularization TEC

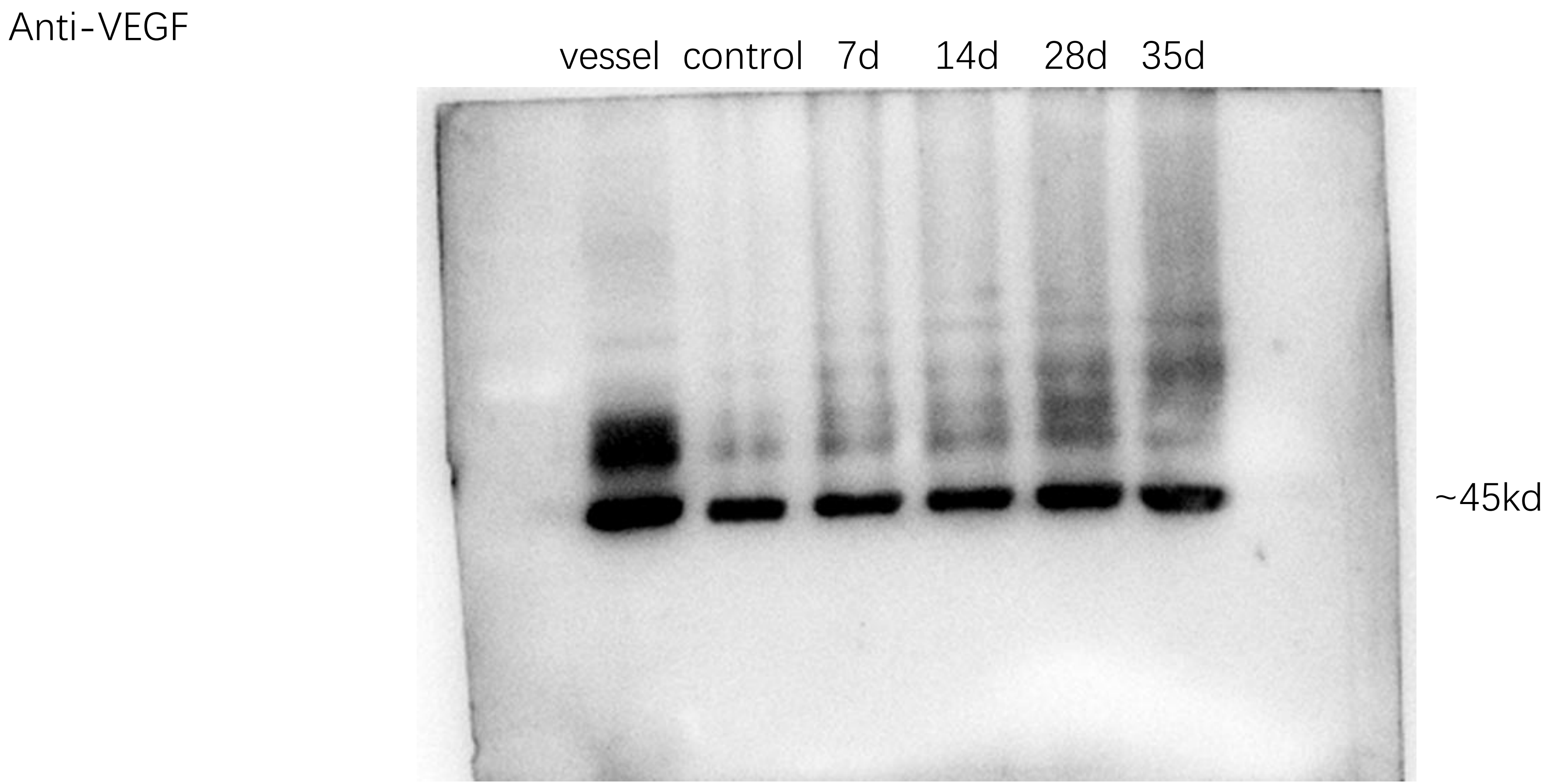

Fig2 C. Relevant data underlying the finding sdescribed in manuscript.

Sample

Vessel: abdominal vessel

Control: Fat pad

7d, 14d,28d,35d:sample from pre-vascularization TEC

Raw data——IHC

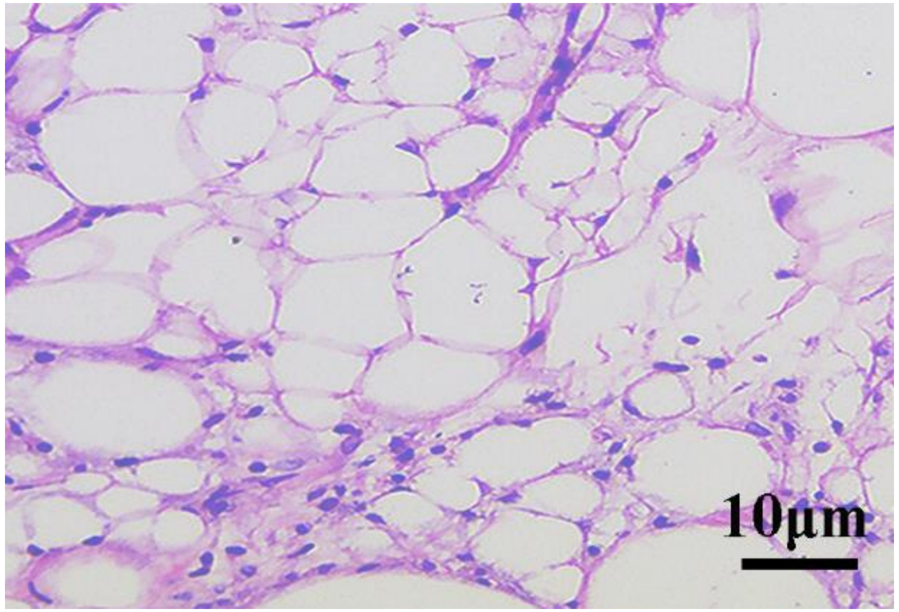

7d

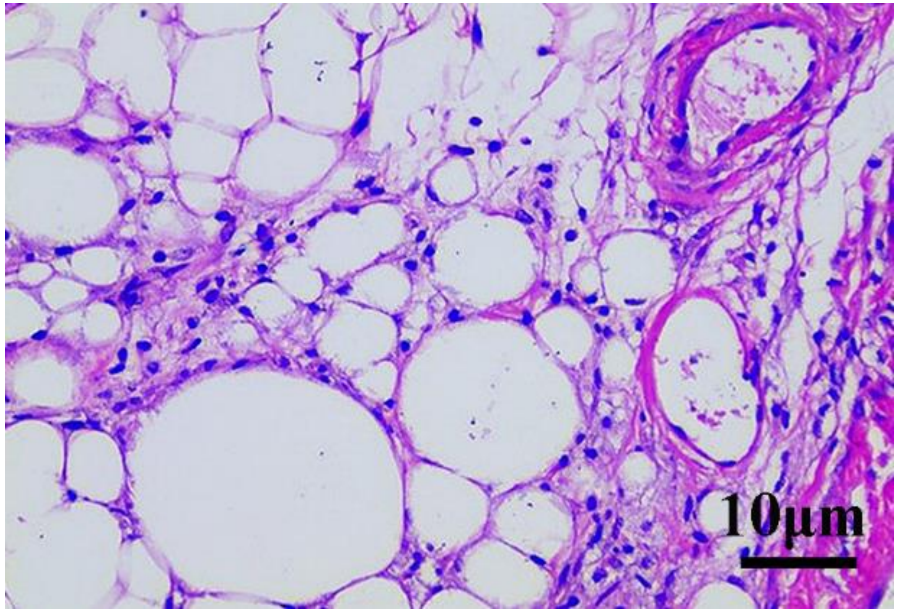

14d

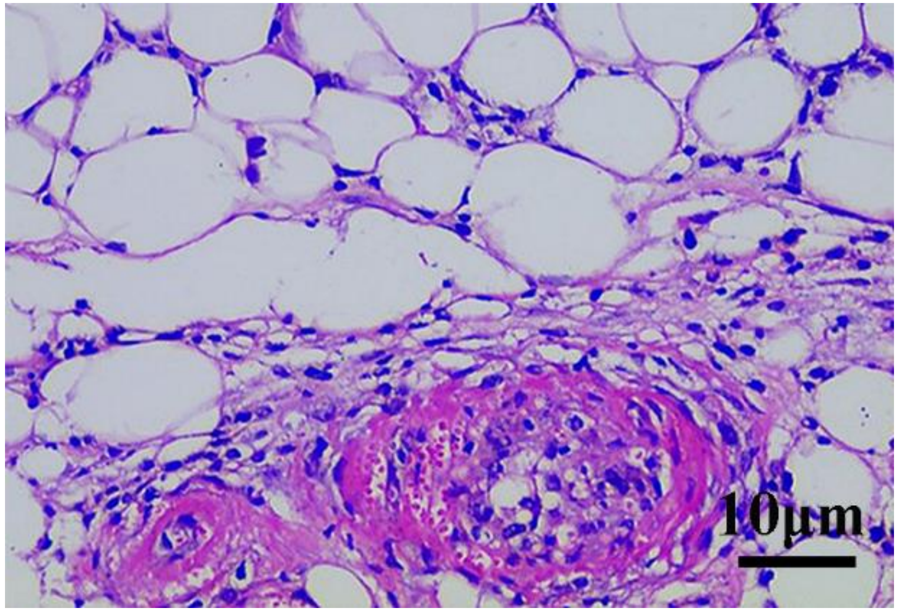

28d

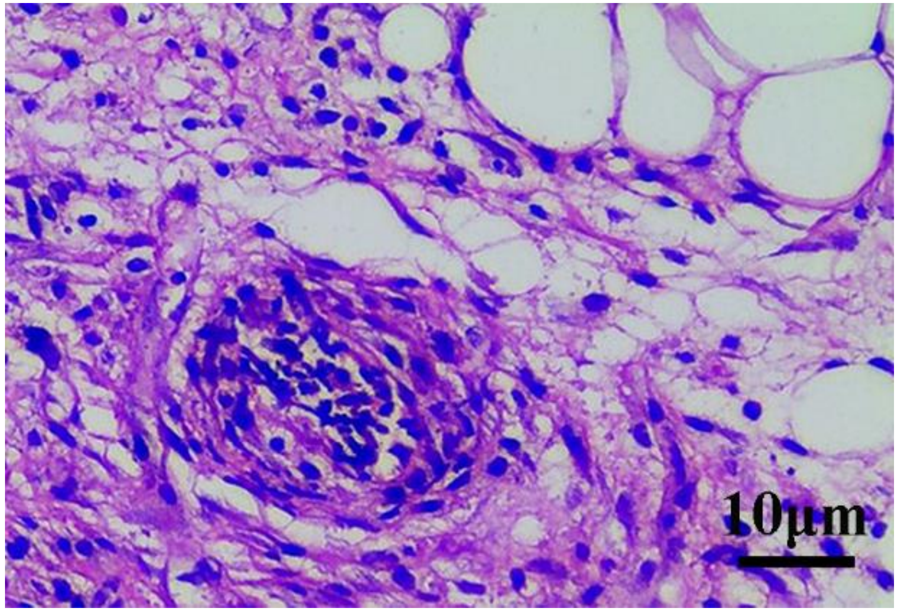

35d

HE

Fig2 A. Relevant data underlying the finding sdescribed in manuscript.

Sample: from TEC

VEGF

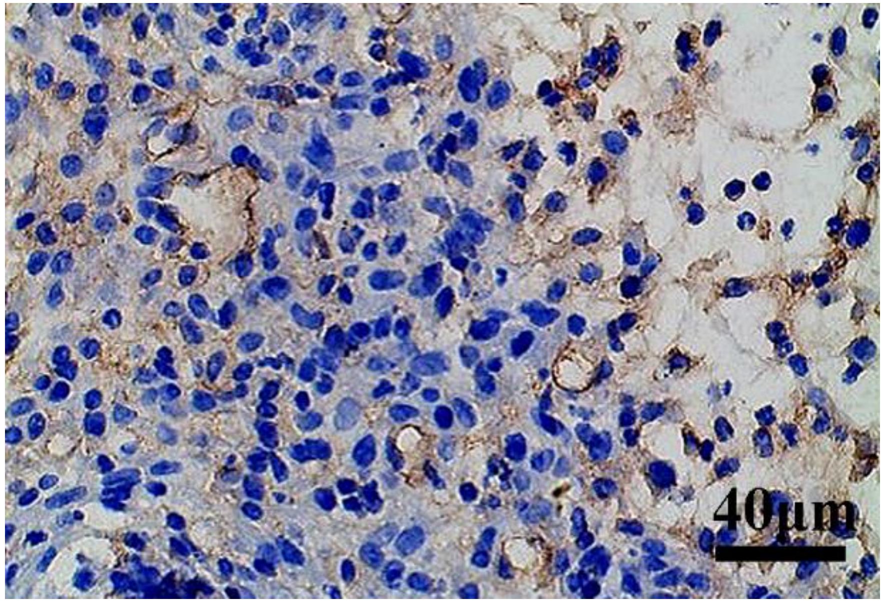

7d

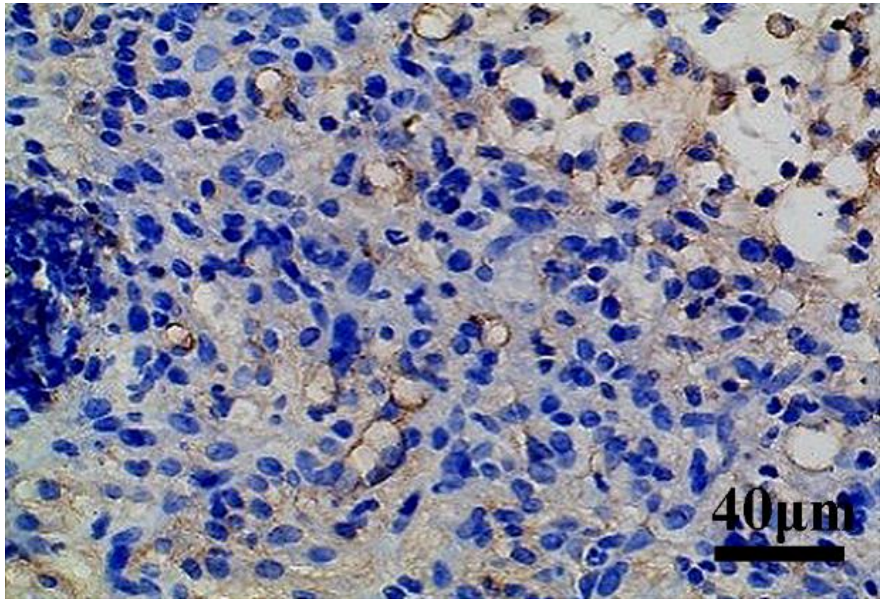

14d

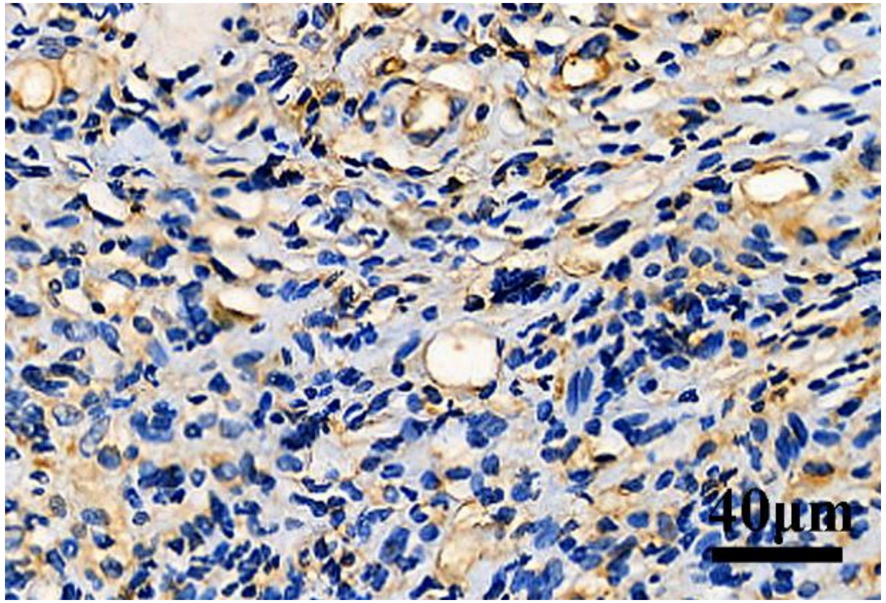

28d

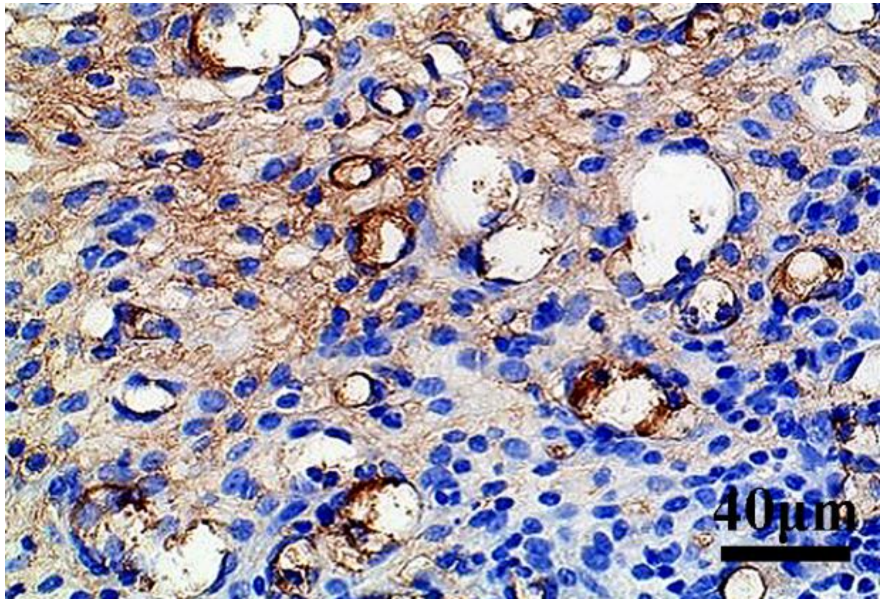

35d

IHC

Fig2 A. Relevant data underlying the finding sdescribed in manuscript.

Sample: from TEC

CD31

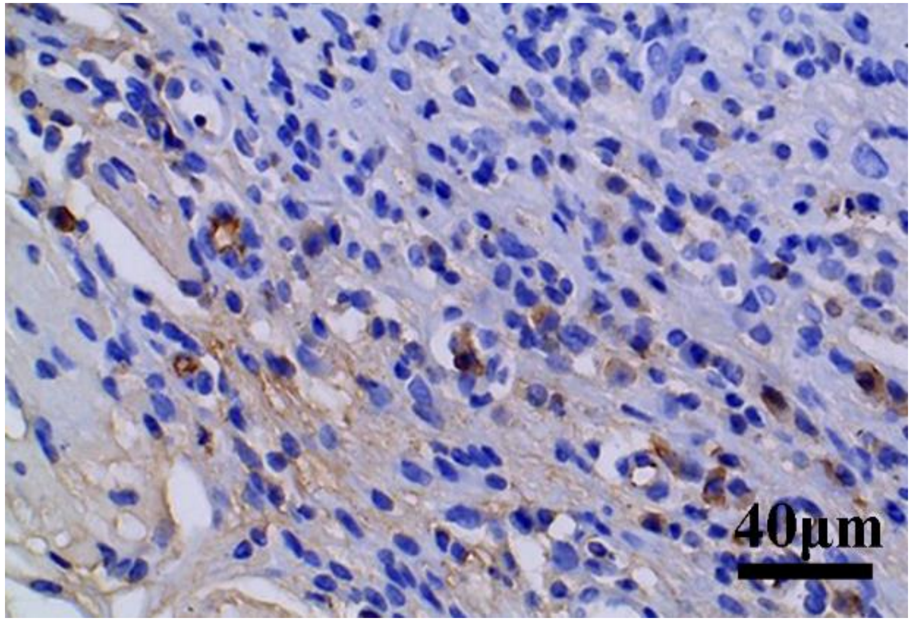

7d

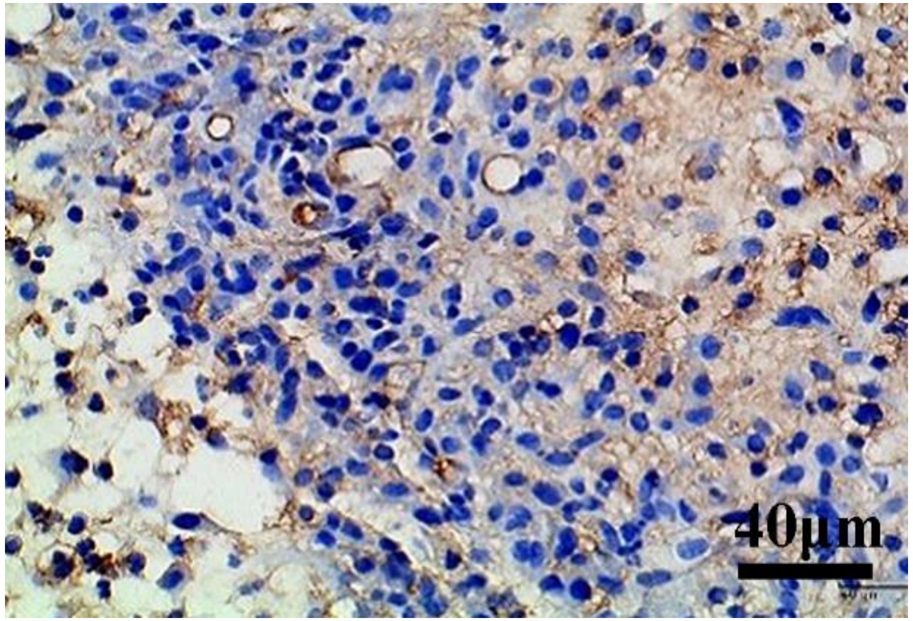

14d

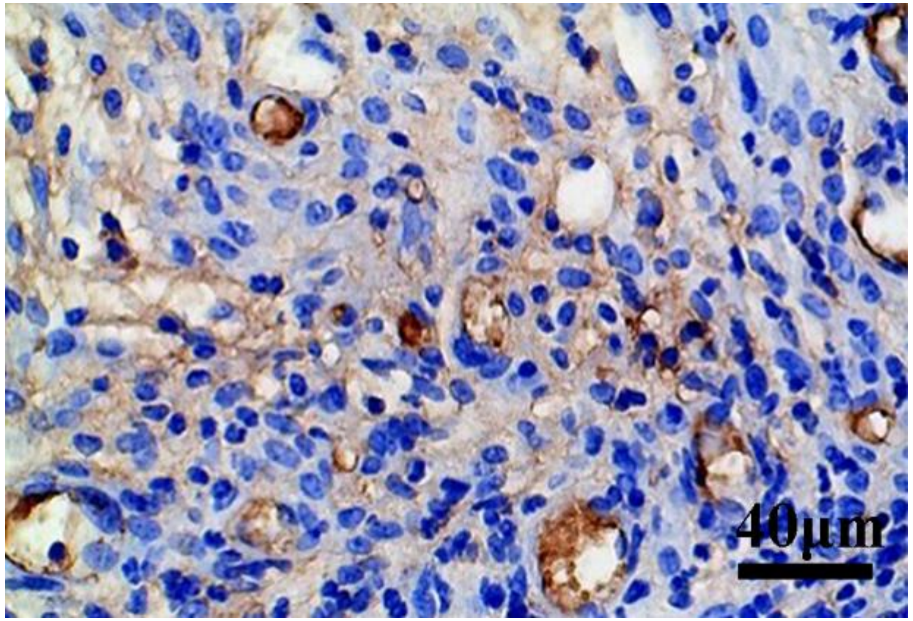

28d

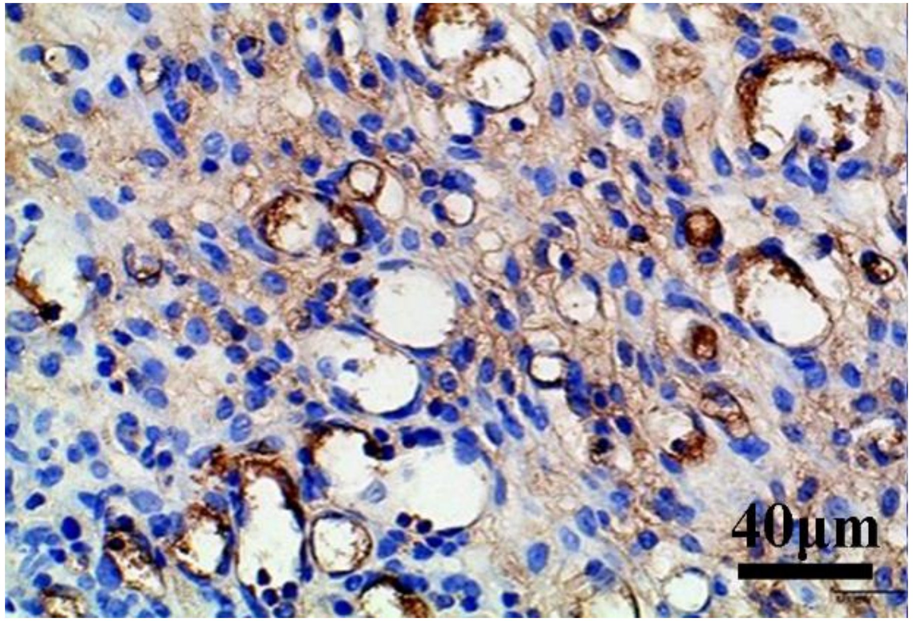

35d

IHC

Fig2 A. Relevant data underlying the finding sdescribed in manuscript.

Sample: from TEC

CD4、CD8

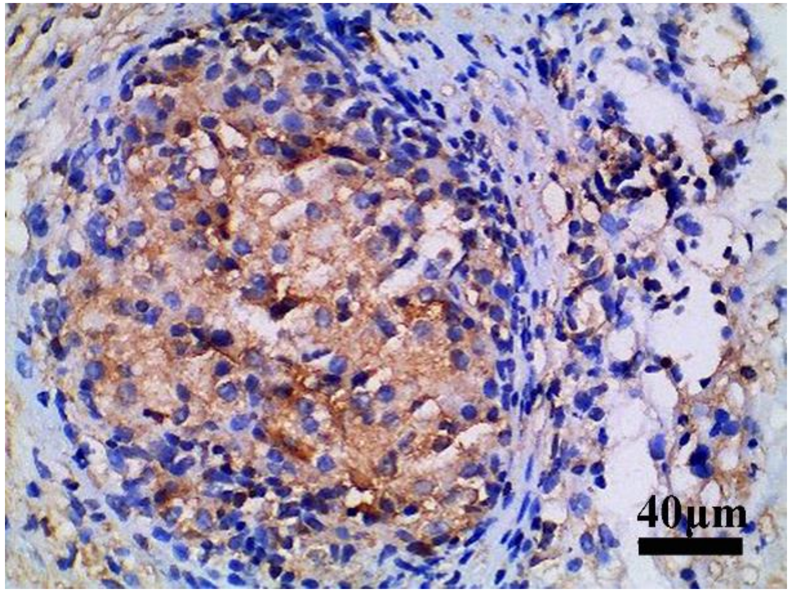

Untreated-CD4

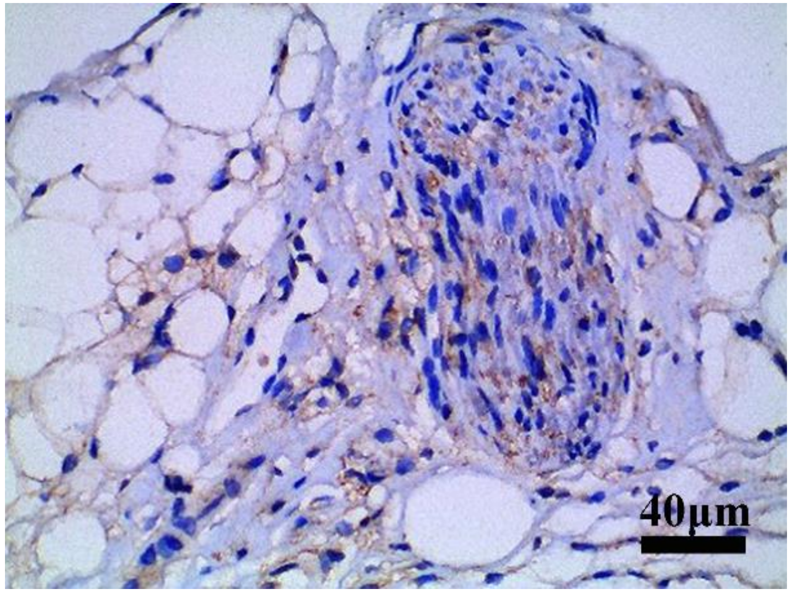

Treated-CD4

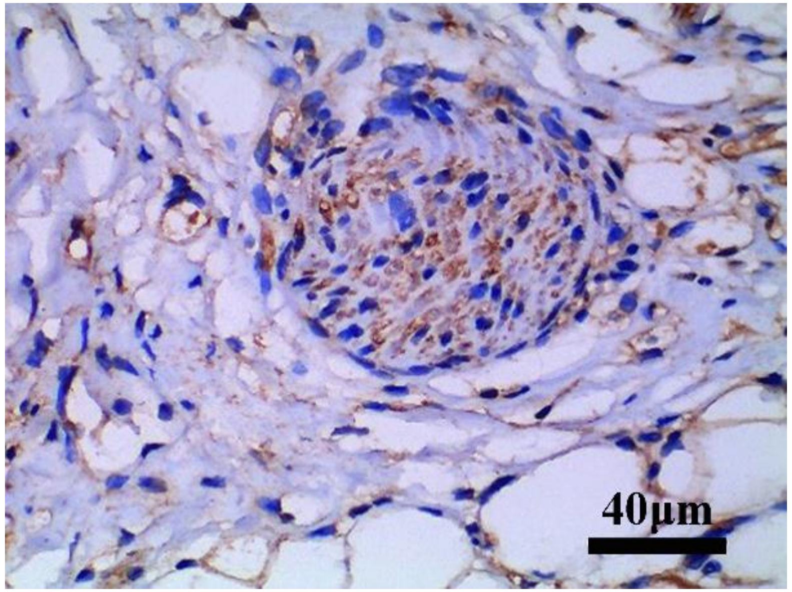

Untreated-CD8

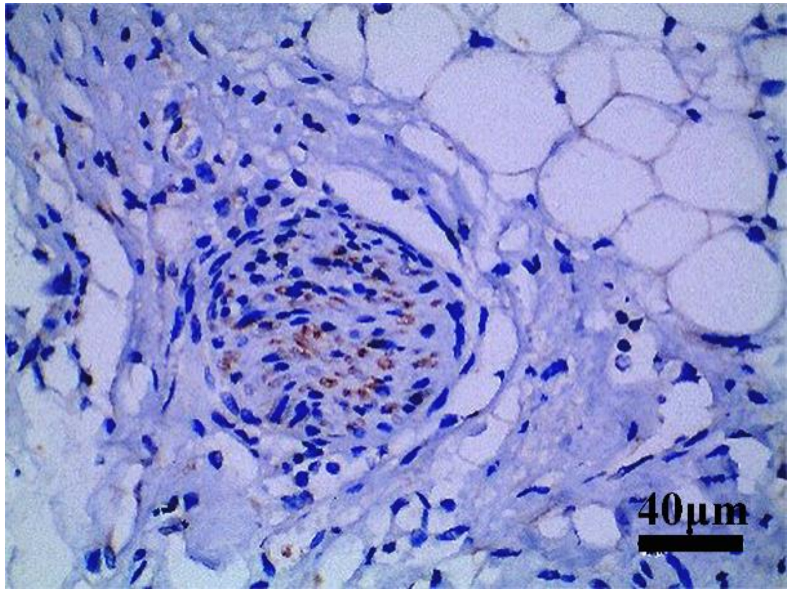

Treated-CD8

IHC

Fig 5A. Relevant data underlying the finding sdescribed in manuscript.

Sample: from TEC

Islets

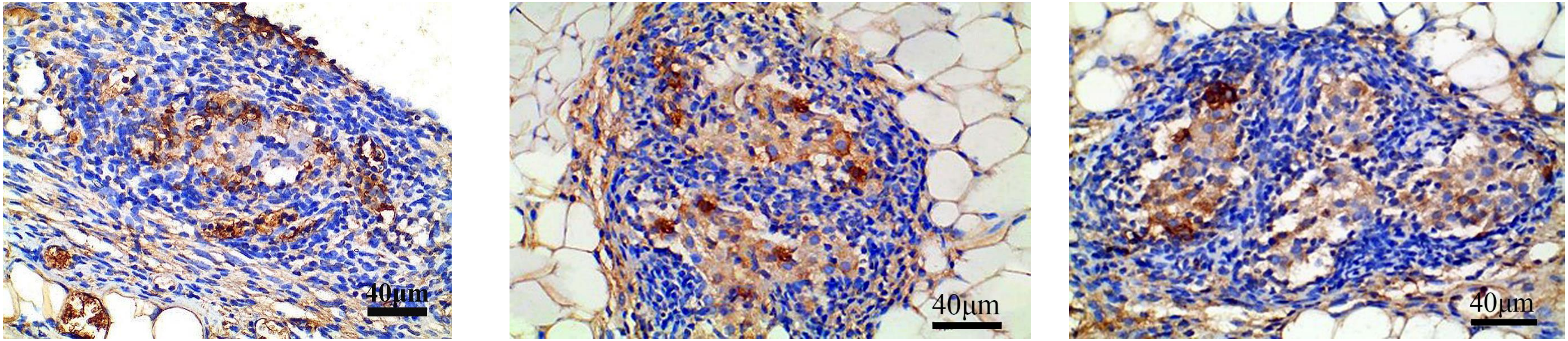

Syngeneic islet **Allogeneic islet** **Xenogeneic islet**

IHC

Fig 5A. Relevant data underlying the finding sdescribed in manuscript.

Sample: from TEC

Raw data——the method of pre-vascularization chamber

the method of pre-vascularization chamber

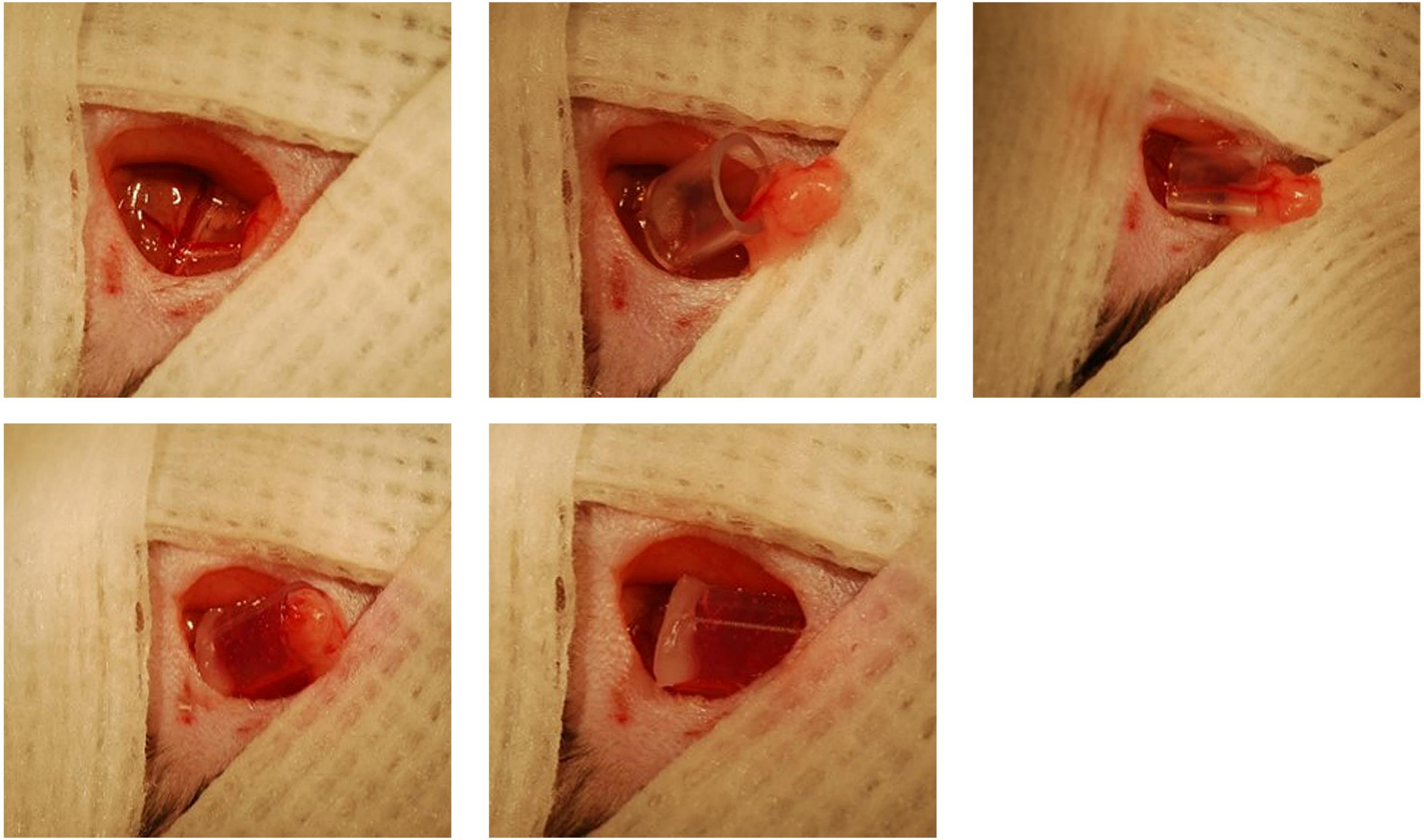

the method of pre-vascularization chamber

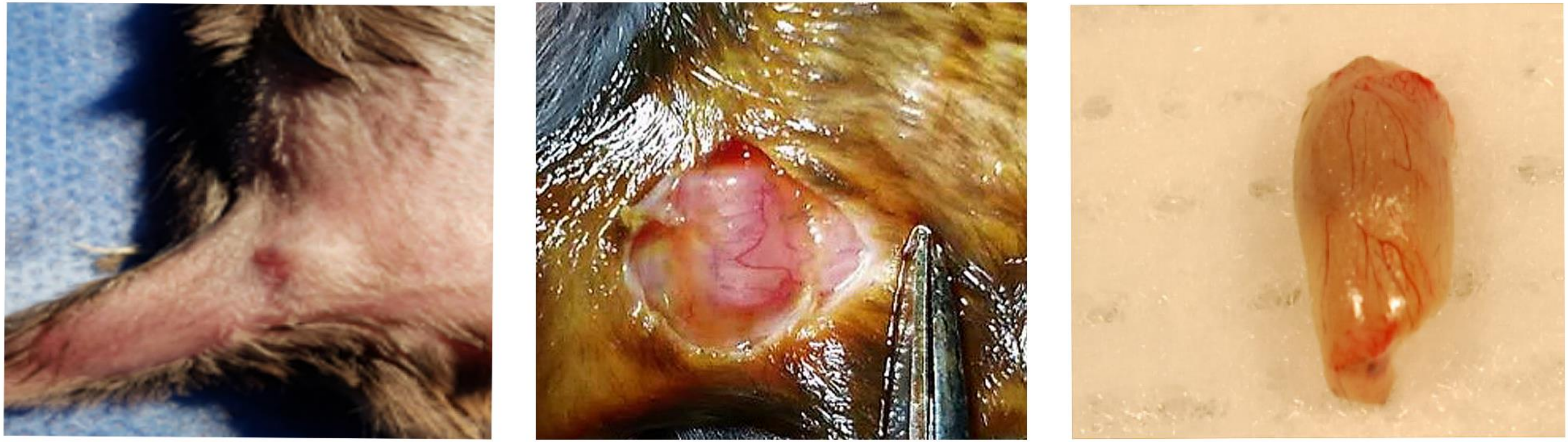

Supplement: S1 Raw data — (PDF) [file pone.0234670.s003.pdf]
