# Supplementary material for: A novel prevascularized tissue-engineered chamber as a site for allogeneic and xenogeneic islet transplantation to establish a bioartificial pancreas
Source: PLoS One. 2020 Dec 3;15(12):e0234670. doi: 10.1371/journal.pone.0234670 (PMC7714105; doi:10.1371/journal.pone.0234670)
Supplement: Attachment — Submitted filename: Response to Reviewers.docx [file pone.0234670.s005.docx]

**Dear editorial-lmanager and Reviewers,**

Thank you for providing the valuable suggestions and comments for our manuscript (ID: PONE-D-20-16218). We have carefully revised the manuscript according to the reviewer comments. The answers to the comments are listed as following.

***Comment 17.*** It is concerning, that only 5 animals are included in each group, and I am surprised to see so little variation, since there normally is significant variation among animals. Raw data should be reviewed. Statistical analysis of samples from 5 animals is not sufficiently robust to draw solid conclusions from. Has power analysis been performed?

***Answer:*** This prevascularized chamber model in murine is really technically demanding and time consuming. And the same model has been also used in islet transplantation in murine groin by different researches [1]. Cronin KJ et.al. used this model to test effects of different materials on revascularization. However, the innovation point in our study is not repeat the previous work which have been proved a successful method to build a prevascularized chamber for islet transplantation, but deepened the work and used this prevascularized chamber model to xenograft transplantation combined with the immunotherapy regime exploited by our research group. Thus, as to a well-documented prevascularized chamber model for islet transplantation, we allocated a small number of valuable model to prove this method could work in allograft transplantation just as that have been well proved by Hussey AJ and Hussey AJ [1], and distributed large number of precious model to test the effect of xenograft transplantation (n=20 in our study). In addition, compared to other works (n=6 in Hussey AJ’s work, and even n=5 in Andrew R Pepper’s work---fig.5 b)[2], the sample size (n=5 in our research) can also prove the efficient of prevascularized chamber in syngeneic and allogeneic transplantation. Thank you again for your careful review and sincere suggestions.

1. Cronin KJ, Messina A, Knight KR, Cooper-White JJ, Stevens GW, Penington AJ, et al. New murine model of spontaneous autologous tissue engineering, combining an arteriovenous pedicle with matrix materials. Plastic and reconstructive surgery. 2004;113(1):260-9. Epub 2004/01/07. doi: 10.1097/01.prs.0000095942.71618.9d. PubMed PMID: 14707645.

2. Pepper AR, Gala-Lopez B, Pawlick R, Merani S, Kin T, Shapiro AM. A prevascularized subcutaneous device-less site for islet and cellular transplantation. Nature biotechnology. 2015;33(5):518-23. Epub 2015/04/22. doi: 10.1038/nbt.3211. PubMed PMID: 25893782.
